# Vinobot and Vinoculer: Two Robotic Platforms for High-Throughput Field Phenotyping

**DOI:** 10.3390/s17010214

**Published:** 2017-01-23

**Authors:** Ali Shafiekhani, Suhas Kadam, Felix B. Fritschi, Guilherme N. DeSouza

**Affiliations:** 1 ViGIR Lab, Department of Electrical Engineering and Computer Science, University of Missouri, Columbia, MO 65211, USA; AShafiekhani@mail.missouri.edu; 2 Division of Plant Sciences, University of Missouri, Columbia, MO 65211, USA; KadamSu@missouri.edu (S.K.); FritschiF@missouri.edu (F.B.F.)

**Keywords:** field phenotyping, robotics, vision, 3D reconstruction, mobile robotics

## Abstract

In this paper, a new robotic architecture for plant phenotyping is being introduced. The architecture consists of two robotic platforms: an autonomous ground vehicle (Vinobot) and a mobile observation tower (Vinoculer). The ground vehicle collects data from individual plants, while the observation tower oversees an entire field, identifying specific plants for further inspection by the Vinobot. The advantage of this architecture is threefold: first, it allows the system to inspect large areas of a field at any time, during the day and night, while identifying specific regions affected by biotic and/or abiotic stresses; second, it provides high-throughput plant phenotyping in the field by either comprehensive or selective acquisition of accurate and detailed data from groups or individual plants; and third, it eliminates the need for expensive and cumbersome aerial vehicles or similarly expensive and confined field platforms. As the preliminary results from our algorithms for data collection and 3D image processing, as well as the data analysis and comparison with phenotype data collected by hand demonstrate, the proposed architecture is cost effective, reliable, versatile, and extendable.

## 1. Introduction

Population increases, climate change, degradation and loss of arable land, and the increasing appearance of new pests and diseases threaten the world’s food supply [1]. Understanding how plants respond to environmental and genetic perturbations is essential to accelerating the improvement of crops and agriculture [2]. High-throughput phenotyping provides an unprecedented opportunity to study the physiological, developmental, and molecular mechanisms that govern the dynamic behavior of plants [3]. However, existing systems that allow highly automated collection of basic phenotypic data for small numbers of plants in the greenhouse fall far short of the need to examine and characterize thousands of plants under real world conditions. Building systems that can collect multi-modal, multi-character data in real time in the field requires integrating plant biology and crop science with robotic vision and computer engineering. These systems must be accurate and reliable, and should provide richer information than the current methods available for automated greenhouse or manual field phenotyping. By doing so, they will help us to link plant genotypes as well as the molecular and eco-physiological responses with the expression of specific phenotypes in response to the growing conditions [4].

In this paper, we present a robotic architecture for high-throughput phenotyping (HTPP) in the field. The architecture consists of two robotic platforms: an autonomous ground vehicle (AGV) for data collection from individual plants; and a mobile observation tower to inspect the entire field and identify specific areas influenced by biotic and/or abiotic stresses. By combining a new hardware architecture, new algorithms, and state-of-the-art sensory devices, these two platforms can be deployed on existing farms, at a much more reasonable cost, specially when compared to alternatives involving aerial surveillance [5,6] or confined field platforms [7].

In the next section, we briefly survey platforms for phenotyping and show cost comparison between our proposed architecture and other approaches in the literature, in special those reliant on aerial vehicles. In that same section, we also summarize the technological challenges in field-based phenotyping, namely: autonomous operation, plant imaging, and crop characterization in outdoor conditions. In the last three sections, we present in detail the proposed robotic architecture, the preliminary results, and conclusions.

It is important to mention here that our goal in this paper is not to investigate current or new traits for phenotyping, let alone to establish their correlation with the physiology, development, or the behavior of plants. Instead, our goal is to show that the architecture, the sensors, and the algorithms for imaging proposed here lend themselves to a reliable, accurate, and fast approach that can be successfully employed to extracting any current or new trait.

## 2. Background

Automatic field phenotyping has been investigated for many years and it can be abundantly found in the literature [8,9,10,11,12,13]. Looking back at some of this early literature, tractors should be regarded as the first ground vehicle to operate automatically in the field [10], even if still in a rudimentary way. In recent years, given the world requirements for high-yielding crops, demand for more fully automated systems is increasing. Those systems include both manned and unmanned, as well as ground and aerial vehicles, all equipped with advanced sensors and sophisticated control algorithms. From those early systems to today’s field robotics, developing reliable sensing technologies, from reliable odometry to sophisticated machine vision, plays a key role in advancing agricultural automation. So, in the next subsections, we discuss the challenges faced by past and future research when it comes to developing a successful high-throughput plant phenotyping (HTPP) platform.

### 2.1. Platforms for Phenotyping

Based on the literature, platforms that are designed for HTPP can be grouped into two categories: those developed for indoor (greenhouse or laboratory) [13] and those designed for outdoor environments (field) [14,15]. Alternatively, platforms may be classified based on whether they collect data from a group of plants or from individual plants.

The majority of efforts in recent years has been placed on the development of HTPP platforms for plants grown in controlled environments (i.e., greenhouses). For instance, in [13], the authors introduced an automated HTPP platform, Phenoscope, to collect data from 735 individual pots brought into imaging stations by conveyor belts. Like other indoor platforms, Phenoscope is able to water, weigh and image plants continuously, allowing for homogenized conditions for all plants. The same is true for the ${\mathrm{Scanalyzer}}^{3DHT}$, made by LemnaTec and reported in [16]. However, this indoor platform also has the ability to capture images in different wavelengths: i.e., from far infrared (FIR) to ultraviolet (UV). The platform is equipped with a system of conveyor belts capable of carrying 600 individual plants, and its imaging systems can capture 9000 images per day [16]. Each plant carries a radio frequency identification (RFID) for individualized data management, watering and nutrient supplementation. Although, greenhouses offer extraction of a large number of features throughout the year, correlation of these features with those of plants grown in the field [17,18] is often unsatisfactory.

Therefore, platforms for field phenotyping with various degrees of automation have emerged over the years [11,14,15,19,20,21]. In [20], for example, the authors introduced a tractor-pulled platform, BreedVision, consisting of various optical sensors, such as light curtain, 3D Time-of-Flight (ToF) cameras, laser range sensors, and multispectral cameras, including RGB. A similar platform was recently presented in [15], with a series of sensors including infrared thermometer (IRT), ultrasonic sensor, and crop canopy sensors: multispectral, NDVI, etc. This combination of sensors allowed the system to collect both architectural and morphological information from plants. However, they employed a vehicle with limited clearance, and hence, limitations on the types of plants to be analyzed could be expected. Also, while the system could cover a reasonably large area at each passage, its limited maneuverability and the fact that navigation was not fully autonomous constrained its throughput and its ability to freely move in the field. In that sense, a faster and more flexible approach is found in [11], thanks to its more autonomous field navigation and its individual plant phenotyping capabilities. Adapting existing equipment is not always a limiting factor. In [14] for example, a field-based HTPP platform was developed using a high-clearance tractor—later referred to as a Phenomobile [22]. Three types of sensors were grouped in four sets and mounted on the front boom of the tractor enabling the simultaneous collection of data from four adjacent rows of cotton plants. Although most phenomobile platforms have a high coverage rate (in this case, 0.84 ha/h), soil compaction is often a main concern [23].

One way of coping with soil compaction and to further increase throughput is to phenotype large groups of plants at once, even if at a lower level of detail—e.g., pixel resolution per plant. In that case, Unmanned Aerial Vehicles (UAVs) are the platform of choice for field phenotyping [24,25,26,27]. However, due to FAA’s regulations and requirements for pre-filing of flight plans, UAV’s limited payload and timely availability, the use of UAVs for field phenotyping is still quite restrictive compared to other approaches [28,29]. One such approach is the ${\mathrm{Scanalyzer}}^{Field}$, by LemnaTec [7]. This platform consists of two 125 m long tracks placed up to 10m apart, with a suspended structure that can be lifted up to 4.5 m in the air. The structure houses sensors with a maximum payload of 500 kg and may include RGB, Near IR, IR, VNIR, Multispectral, and Fluorescence intensity cameras, as well as NDVI, carbon dioxide, temperature, light intensity, and wind speed sensors. However, it can only cover a small area (0.12 ha) and is not easily transferable to other sites—the installation of the tracks being the major financial and logistic undertake in the use of this system [9].

It was exactly with these criteria in mind—speed, cost, availability and payload—that we developed our architecture. In Table 1, we summarize the costs involved in the use of an UAV-based solution for phenotyping versus our proposed solution. The commercial UAVs surveyed for Table 1 were: Draganfly X4-P and Commander; Allied Drones HL11 Atlas; Steadi Drone Vader HL and Vader X8; SciAero 4Scight; Xactsense Max-8; and AEE F100. Unfortunately, we did not have access to the cost of the LemnaTec’s ${\mathrm{Scanalyzer}}^{Field}$, but it is expectedly more expensive, specially if considered dollars per acres covered. In Section 3.2, we expand on this comparison by showing the advantages of the proposed architecture in terms of the other criteria—i.e., throughput, payload, and sensor capabilities.

### 2.2. Navigation in the Field

Autonomous navigation refers to the ability of a robot to move within its habitat automatically. In order to achieve that, motion control and localization algorithms are required to accurately determine the robot’s position in the environment and to compute the path through obstacles—in our case the field and plants, respectively. This is a quite difficult type of navigation referred to as *outdoor navigation in unstructured environment* [30]. Autonomous navigation using differential GPS is frequently used to mitigate some of the challenges of navigation in unstructured environments. However, vision-based guidance is getting more attention as it can potentially reduce costs, handle dynamic situations and simplify installation, while it can achieve precision comparable or even better than from Global Navigation Satellite Systems (GNSS). In that sense, sensors such as LiDAR and RGB cameras along with new algorithms for either 2D or 3D dynamic navigation indeed provide increased flexibility in such a unpredictable environment [21,31,32]. For instance, in [33,34], the authors developed an algorithm for detecting tree trunks using data fusion from camera and laser scanner. The system developed could navigate throughout a fruit orchard .

In another system, [35], the autonomous vehicle used a method to track crop rows using the surrounding texture of the agricultural field. The method worked even in extremely varied appearance of the field, such as under day and night lighting conditions. As data collected from the field had noise due to uncertainty in the environment, a proper manipulation of data was required. Finally, Hiremath et al. proposed a novel probabilistic sensor model for a 2D range finder (LiDAR) and also RGB camera for robot navigation in a maize field [12,36].

In this research, most of the challenges in navigation were mitigated by the use of a semi-autonomous approach. In the future, a completely autonomous method relying on 3D imaging, GPS, and LiDAR will be employed.

### 2.3. Computer Vision in Plant Phenotyping

In order to understand plant adaptation to the environment and management practices, architectural phenotypes such as height, width, leaf area, leaf angle, leaf color, etc. are very important [37]. Traditionally, these traits are measured by hand, consuming an enormous amount of time. To date, computer vision has already made an impact in speed and volume of plants phenotyped (i.e., high throughput), specially when it comes to phenotyping in growth chambers and greenhouses [13,17,38,39,40,41,42]. Also, 3D imaging of plant shoots and roots [43,44,45] are becoming the standard in storing all possible details from plants—i.e., details with hitherto unknown value, but which can prove useful in the future. Indeed, while 3D imaging in the field may still have a long way to go vis-a-vis their greenhouse counterparts, some systems, such as in [46], are already causing great impact. In this work, the system relied on a structure from motion algorithm [47] over a sequence of images of the crop rows to build 3D models and estimate plant height and leaf area.

### 2.4. Plant Canopy Characterization

Crop canopy characteristics critically influence yield formation [48]. In fact, important traits such as plant height, weight, volume, biomass, shape, color, temperature, light absorption and potentially many others can be obtained from the simple observation of the canopy as a whole. In that case, thermal, multi-spectral and hyper-spectral imaging from either airborne or remote sensing (satellite) can play a greater role in plant phenotyping in the field. Unfortunately, the cost and availability of these systems may pose a large burden on research and still lack the necessary resolution. In that sense, while some researchers invest in larger vehicles to increase payload, optimize volume and type of data acquired on a single flight [49], other systems rely on multiple micro-UAVs to achieve higher availability [50] and still perform canopy characterization. In [51], for example, a simple, but ingenious technique for measuring the height of plants using micro-UAVs was proposed. In that system, a laser scanner was mounted onto the micro-UAV to estimate plant height by measuring the difference between the ground and the top of canopy. Indeed, despite the persisting issues with cost and availability, successful aerial systems for canopy characterization abound, and it would be hard to survey all in this paper.

## 3. Proposed Phenotyping System

As evidenced by the literature, advances in sensing technology can make plant phenotyping more reliable, easily available, and accurate. Consequently, robots that can deliver and operate those sensor platforms in the field are in high demand. In fact, without robots that can operate such sensors automatically and with high availability in collection of both structural and environmental characteristics, true HTPP is impossible. So, in our research, we developed two phenotyping platforms for in-situ characterization of plant responses to changes in their environment (e.g., management practices, drought, flood, heat, etc.). These platforms consist of two robotic systems: a mobile, observation tower, *Vinoculer*, for canopy characterization and general inspection of the crop; and a ground vehicle, *Vinobot*, for individual plant phenotyping.

**Figure 1:**
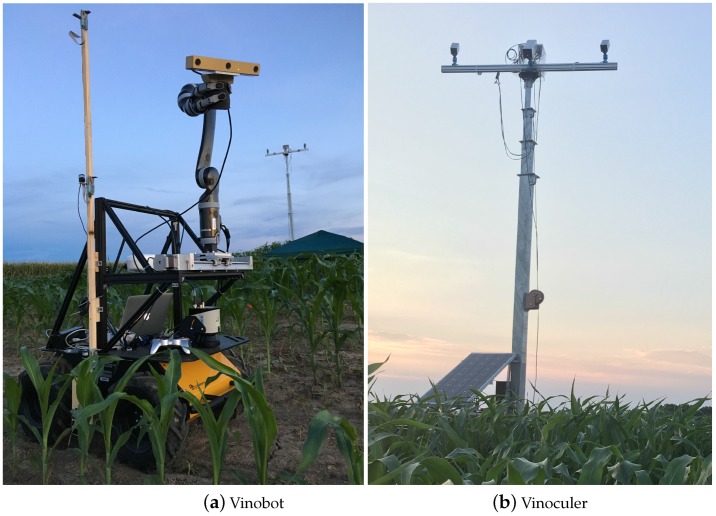
The proposed platforms for high-throughput phenotyping in the field deployed at the Bradford Research Center: (**a**) ground vehicle, Vinobot; and (**b**) observation tower, Vinoculer shown at 15 ft (4.5 m) high.

Figure 1a,b show the Vinobot and Vinoculer in a field at the Bradford Research Center near Columbia, Missouri, USA. In the next section, both systems will be introduced in more details.

### 3.1. The Platforms

The first platform, Vinoculer, for ViGIR-Lab (Vision-Guided and Intelligent Robotics Lab, Columbia, MO, USA) Phenotyping Trinocular Observer, is a mobile observation tower equipped with a 360-degree robotic vision system that can oversee a large area of the crop using two (stereo) RGB cameras and one IR (thermal) camera. The main purpose of this system is to detect regions of the crop under stress, and deploy the ground vehicle for further investigation.

The ground vehicle, or Vinobot for ViGIR*-Lab* Phenotyping Robot, is responsible for phenotyping plants individually. That is, the Vinobot moves around the field and collects data from each plant, on a regular schedule or by demand. The Vinobot consists of multiple sensors, such as for 3D imaging, temperature, humidity, light intensity (PAR), etc. On the Vinobot, we also included a differential GPS, a robotic arm, a LIDAR, and other support equipment for autonomous navigation and operation of the phenotyping sensors.

#### 3.1.1. Vinobot

**Figure 2:**
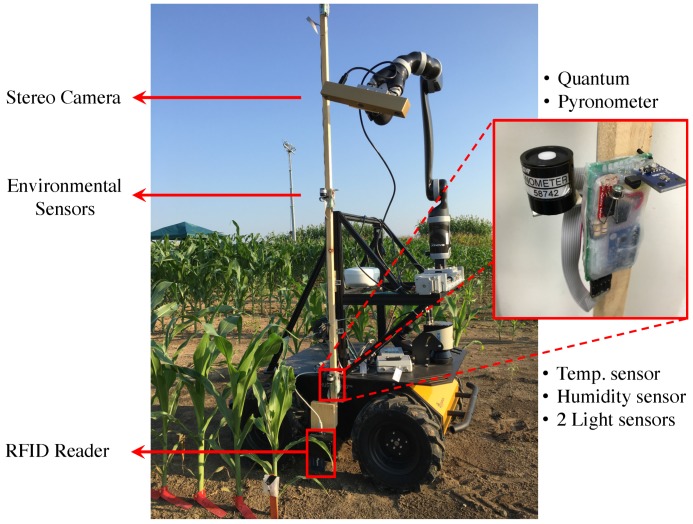
Hardware components of Vinobot.

As just mentioned, Vinobot is a mobile platform equipped with a range of sensors designed to fulfill both the phenotyping tasks and its autonomous navigation needs. Figure 2 depicts the equipment installed aboard the Vinobot, which will be explained in detailed next.

#### Hardware

Vinobot was implemented around the Husky A-200 from Clearpath. The hardware components of the Vinobot are shown in Figure 2. A linear slide at the front of the robot guides a robotic arm (${JACO}^{2}$ from Kinova), which was mounted on the slide to improve lateral reach. The purpose of the robotic arm is to allow for multiple sensors to be handled by the robot. Currently, a BumbleBee XB3, by PtGray, is handled by the arm to perform 3D imaging. In the future the robotic arm will also handle clip-on sensors such as gas exchange analyzers and SPAD meters to quantify additional phenotypes.

In terms of environment, three sets of temperature (TMP36), humidity (HIH-4030), and light intensity (TSL2561, LI-190 SA, and LI-200 SA) sensors were mounted along a vertical bar at three different heights. The three sets perform measurements close to the ground, at mid-plant height and near the top of the canopy. While the robotic arm could chose between multiple sensors and it could easily position a single set of environmental sensors at any height, the use of a vertical bar with the three sets already mounted at three heights was chosen to increase throughput. Also, in terms of light intensity, four sensors were added at the same three positions on the bar: a Pyranometer at the top and a Quantum at the mid-plant and ground levels; and a Luminosity and photo diode at all three levels—the magnified subimage in Figure 2 shows the sensors in detail.

As for plant characterization, a trinocular camera, BumbleBee XB3, was used as already explained above—see Figure 2. Once again, the purpose of the robotic arm is to move the camera around individual plants, allowing for multiple viewing angles and resolving any potential occlusions while imaging the plant.

Various other equipments were contained within the Vinobot, including an on-board PC (Mini-ITX Single board), GPS (Novatel Smart6-L), LiDAR (Sick LMS 151), long-range WiFi, ADCs, Arduinos UNO, etc. These devices are mostly required for the navigation task, but they may also provide support for phenotyping. At this stage of development, one sensor plays an important role in identifying the individual plants: a RFID reader (PN532). This reader is used to detect RFID tags which are placed by each plant, along the rows of the crop.

#### Software

**Figure 3:**
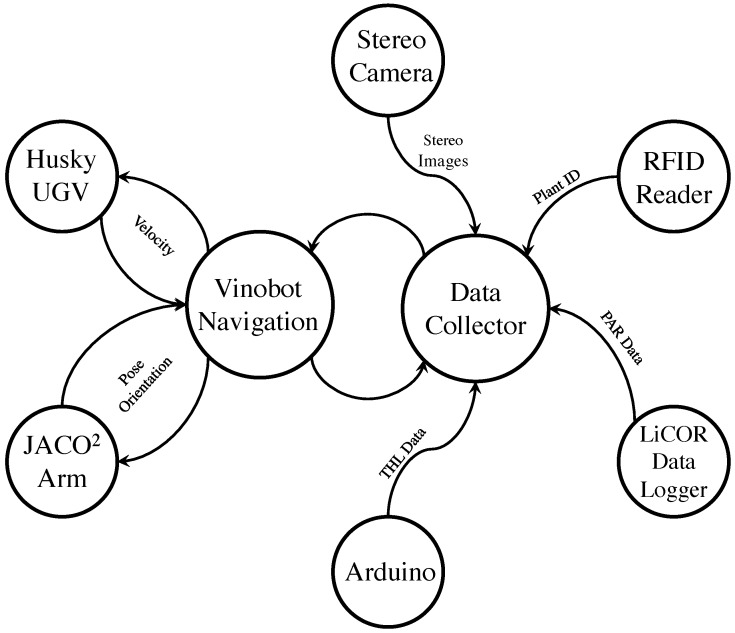
High-level software architecture of the Vinobot.

The software architecture of the Vinobot was made possible by the Robot Operating System (ROS) [52]. Each software module, thread or task in ROS was encapsulated in what is referred to as a node, and ROS provided the mechanisms for interprocess communication through the nodes. There are eight high-level nodes and many more lower-level nodes in the current implementation of the Vinobot. Figure 3 presents the high-level nodes, starting with the Navigation node, which is under development and it currently allows for semi-autonomous navigation. That is, once aligned with the rows, Vinobot can move autonomously, stopping at each plant as this is detected by the RFID Reader node. At that point, the robot performs all the environmental and architectural data acquisition entirely autonomously. As the name implies, the JACO node controls the $JACO^{2}$ arm to move the stereo camera to predefined positions and orientations. The Data Collector node gathers all data from the sensor nodes and saves them under a unique ID associated with the detected plant. The Arduino node publishes temperature, humidity and light intensity (photo diode) data from the three sets of environmental sensors mounted on the vertical bar. Finally, the LiCOR Data Logger node publishes solar radiation flux density and Photosynthetically Active Radiation (PAR) data from the Pyranometer and Quantum sensors, respectively.

The process of moving along the row and collecting data for each plant was repeated until the robot covered the designated area. At that point, a human operator controlled the robot to align it with the next row, and the process of data collection was restarted. In the next version of the system, this process will be completely autonomous, with the designated area to be covered being provided by regular scheduling or by the Vinoculer.

#### 3.1.2. Vinoculer

Vinoculer is a portable observation tower mounted in the center of a field. It is capable of turning 360 degrees while capturing data from a large area. It was equipped with stereo RGB and IR cameras in order to perform measurements such as volume, leaf area, biomass, height, growth rate and other canopy characteristics. Together, the Vinobot and Vinoculer are capable of collecting data from a large area at the canopy and individual plant level, as will be explained later.

#### Hardware

**Figure 4:**
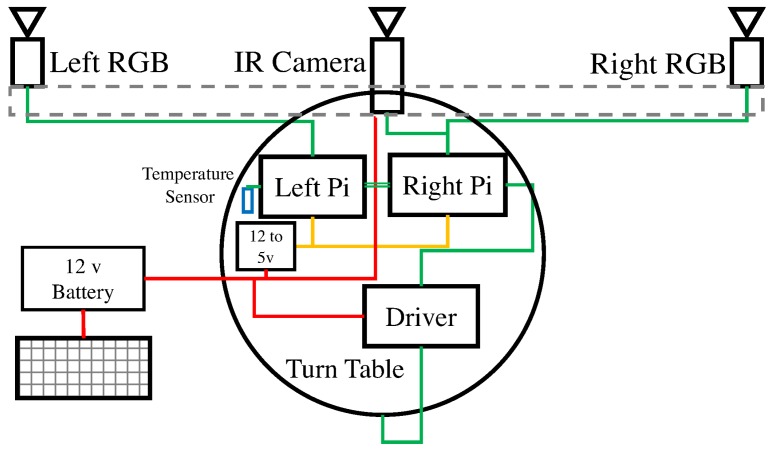
Hardware architecture of the Vinoculer.

The Vinoculer was equipped with two RGB-spectrum cameras for stereo vision (Grasshopper3 by PointGray), an IR camera (Flir A625), a 360° turntable with an accuracy of 0.1°, a temperature sensor, and WiFi—see schematic in Figure 4. This equipment was mounted on a telescopic and easily movable tower, which could be elevated to heights in the range of 3 m to 10 m. Given the characteristics of the current lenses (8 mm), the tower provided Vinoculer with a viewing area with 30 m diameter, when deployed at maximum height, but lenses with shorter focal lengths could be used to increase the viewing area.

The two 12Mpixel RGB cameras were placed on a horizontal bar to provide a stereo baseline of approximately 1.7 m—the IR camera was mounted at the center, between the two RGB cameras. The turntable rotated the set of cameras with respect to the base and the Vinoculer was programed to image the field at regular intervals—as often as every 5 min and in 0.1° steps. Two Raspberry-Pi’s V3 controlled the turntable and the cameras. A total of 64 GB of memory stored data for up to 10 consecutive days, considering a rate of 36 sets of images (RGB + IR) being collected every 30 min and at 4Mpixel resolution. One temperature sensor was added to measure air temperature. The entire system was powered from the ground by 12 V lead-acid batteries, which were charged through solar panels, but could last 2 days without sunlight. The entire system was weatherized to tolerate typical summer conditions in the Midwest of the USA—i.e., over 100 °F (37.8 °C) temperatures, rain, and strong winds (50 mph or 22.3 m/s on record for the period of deployment).

#### Software

The software of the Vinoculer was simpler than the one for the Vinobot. It consisted of a single thread running on the Raspberry Pi’s (RPi) to control the turntable at regular intervals and store the data. The Left RGB camera and air temperature sensor were connected to the Left RPi, while a second RPi controlled the Right RGB and the IR cameras. The Right RPi also controlled the turntable. All cameras were synchronized within 1 ms to collect images simultaneously. As explained in Section 4.2.1, the cameras were calibrated using a specially-designed calibration pattern—with a multi-material surface to allow for the interdependent calibration of RGB and IR cameras.

### 3.2. Advantages over Other Systems

Our architectural design focused on maximizing accuracy, throughput, and payload, while minimizing cost and downtime. So here, we present a comparison between the components of the proposed architecture and other systems in the literature. Our goal is to demonstrate that when accounting for all characteristics above, our system has clear advantages.

As mentioned earlier, one way of achieving a minimum throughput of hundreds of plants per hour to be considered a HTPP system [38] is to perform aerial imaging using UAVs. So in this section, we first compare the Vinoculer with a typical UAV-based system reported in [49]. Table 2 summarizes this comparison mainly in terms of payload, availability, total area covered, and max wind speeds. As the table shows, our system compares favorably in all but one of these criteria: total area covered. But one aspect that is not indicated in the table is the cost to achieve the area covered by a UAV. In that case, we refer back to Table 1, where the cost of 8 commercially available UAVs can reach US$80,000, compared to just $5000 of the Vinoculer.

Next, we considered typical field-based systems that perform phenotyping of groups of plants without resorting to UAVs. In this case we assumed both Vinoculer and Vinobot collect only groups of plants, and Table 3 summarizes the advantages of the combined platforms with respect to two other systems: the “Phenomobile” in [14] and the ${\mathrm{Scanalyzer}}^{Field}$ in [9]. We call the attention of the reader to the fact that the combined Bytes/h of our architecture is comparable to the commercial ${\mathrm{Scanalyzer}}^{Field}$, for a much lower price and without the confinement to a specific area, since both of our platforms are mobile.

Finally, in Table 4 we compare phenotyping platforms that can performed detailed, and hence time consuming scans of individual plants. Once again, while the perfomance of our platform falls below one of the systems in the literature, it still performs much higher than the other system and it does so in outdoor conditions.

## 4. Experimental Results and Discussion

**Figure 5:**
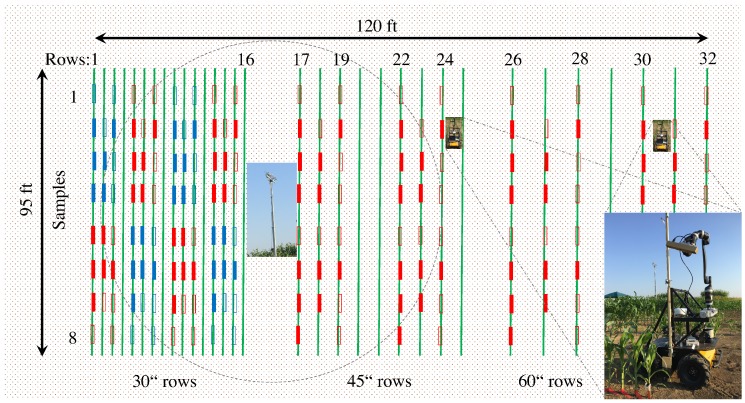
Field configuration at the MU Bradford Research Center. Small vertical boxes represent 10 plants, with sorghum marked by blue and corn marked by red boxes. Full boxes indicated plants selected for destructive sampling.

Various experiments and data collections have been performed at the University of Missouri Bradford Research Center. Experiments were carried out from June to September 2016, using two different fields. In this paper, we will focus on experiments conducted in one of the fields where maize and sorghum were planted in rows with either 30, 45, and 60″, i.e., 76, 114, and 152 cm spacings between rows. Figure 5 shows the spatial configuration of the rows and plant species distribution, as well as the location of the Vinoculer in the center of the field, and two illustrations of the Vinobot as if moving between two rows. Each small vertical box in the Figure represents 10 plants, usually selected for destructive sampling—i.e., for manual phenotyping and hence, the source of ground truth data. The filled boxes indicate the plants actually used in the experiments in this paper. The red boxes mark the areas with maize and blue boxes those with sorghum. Ground truth data were collected from all row spacings and related to Vinoculer-based data obtained for the 76 and 114 cm row spacings, and to Vinobot-based data obtained for the 114 and 152 cm row spacings. This sampling method is summarized in Table 5. Sample data collected by both the Vinobot and the Vinoculer were made available to the public and can be found at [53].

### 4.1. Vinobot

Using the Vinobot sensors, a series of measurements and derived data were obtained and are presented next. These data include: full 3D Models of the plants for further/future feature extraction; plant height; Leaf Area Index (LAI); multiple light measurements; and air temperatures (presented in Section 4.3).

#### 4.1.1. Traits from 3D Reconstruction

**Figure 6:**
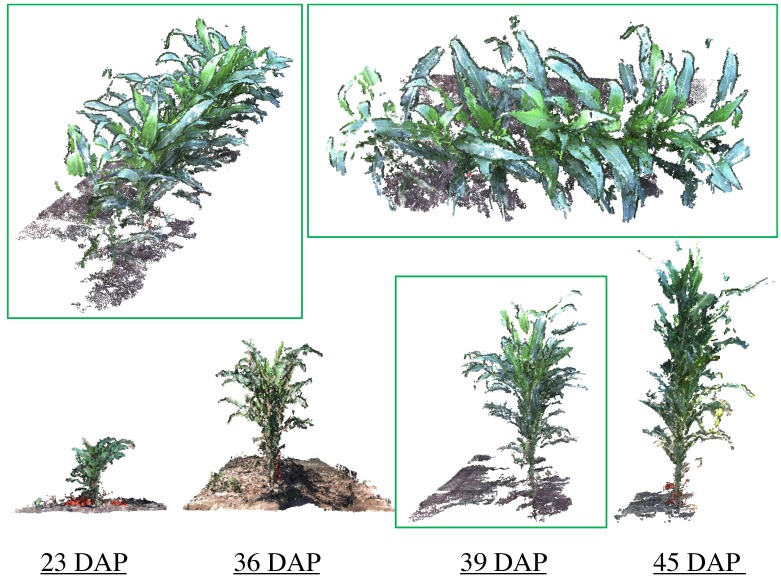
Typical examples of 3D reconstructed plants at four different DAP (days after planting) using stereo images collected by the Vinobot in rows 17 and 18 in Figure 5.

As mentioned earlier, the Vinobot arm is capable of handling different sensors, including a RGB trinocular camera. The images collected by this camera have been used to create 3D reconstructions of the plants. Figure 6 shows typical 3D models of plots during the growing period—i.e., at four different stages of plant growth. An algorithm based on the Visual Structure From Motion, or VisualSFM [54] has been used to create the dense reconstructed models presented here. Many traits can and will be extracted in the future from the dense 3D models already created, however, only two traits will be presented in this paper: height and LAI. The reader is invited to check the quality of these dense 3D models from [53].

#### 4.1.2. Plant Height

**Figure 7:**
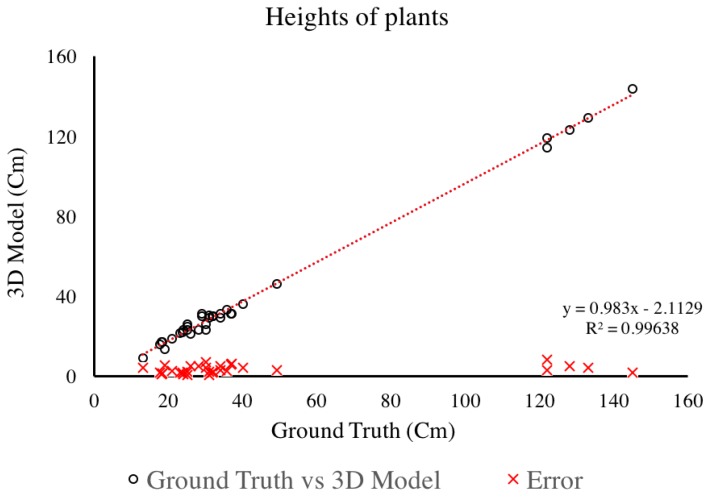
Relationship of plant height measured manually and plant height extracted from the 3D Model created using RGB images captured by Vinobot.

The 3D reconstructions can be used to extract many features, including plant height. For this study, plant height was measured as the distance from the ground to the collar of the top leaf. In Figure 7, we present the relationship between plant height measurements conducted manually and those extracted from the 3D reconstruction. As Figure 7 shows, the RMS error noted for this comparison is less than 0.5 cm, which is smaller than the acceptable accuracy from manual measurements—i.e., human error. The gap in data between 50 to 120 cm, was due to the fact that the manual phenotyping focused on plant early development, while the later measurements were extracted only for the purposed of further validating this system.

#### 4.1.3. Leaf-Area Index

Leaf Area Index (LAI) is commonly used to assess crop growth because it provides biologically relevant canopy-level information. It is defined as the ratio between the total area occupied by the leaves and the area available per plant. This ratio can be obtained manually from the ground [55] or from airborne systems [56]. In fact, airborne LAI measurements are often not very reliable and most often they equal “green leaf area visible from above” more closely than real LAI. Manual LAI is some times obtained by measuring the area of all leaves individually, which is a time consuming process that usually requires destroying the plants. Other quicker and non-destructive methods used to determine LAI involve calculating the portion of green area from fish-eye photography or alternatively the portion of light under the canopy, i.e., that passes through the canopy (e.g., LAI-2000 to determine LAI [57,58]). Although non-destructive methods have the advantage of being fast and inexpensive, they usually underestimate the actual value of LAI due to the common overlaying of leaves.

**Figure 8:**
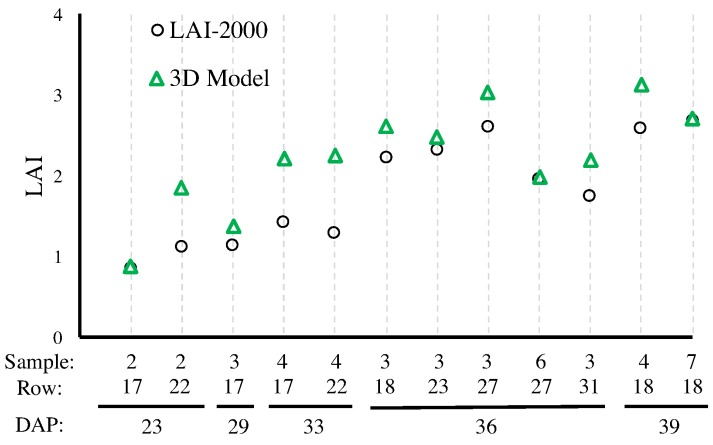
Comparison of LAIs obtained by LAI-2000 and Vinobot using 3D reconstruction of the leaves. The horizontal axis shows the number of samples collected per row; the row number from Figure 5; and the days after planting (DAP) for each observation.

In this research, we used two methods for estimating LAI through 3D reconstruction: from the Vinobot, described next, and from the Vinoculer described in Section 4.2.4. For the Vinobot, the single-sided surface of the leaves was computed for each leaf and then summed over all leaves, with final LAI determined as follows:

$$\mathrm{LAI}=\frac{\sum S_{i}}{A}$$

where $S_{i}$ is the area of i-th leaf and *A* is the area of ground available to the plant as determined based on plant density. Figure 8 compares LAI values measured by the LAI-2000 versus the ones obtained by the Vinobot. As expected, the values obtained from the LAI-2000 are consistently lower than the LAI obtained by the Vinobot—an observation that will be repeated for the Vinoculer and can be explained by the undetected overlaying of leaves in traditional methods.

#### 4.1.4. Light Exposure

**Figure 9:**
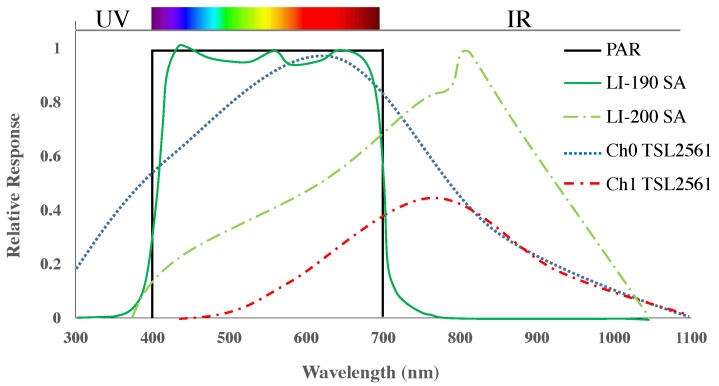
Wavelength sensitivity for the LI-190 SA, the LI-200 SA and the two outputs from the TSL2561.

The Vinobot was equipped with three types of light sensors: a LI-COR LI-190 PAR Quantum sensor (or Photosynthetically Active Radiation Quantum); a LI-COR LI-200 Pyranometer; and a TSL2561 photo diode with two different frequency responses – i.e., for a total of four wavelength sensitivities. Figure 9 shows the different wavelength characteristics for each of these sensors. A factor of 0.0185 was applied to convert Lux to Photosynthetic Photon Flux Density, or PPFD (μmol·$m^{-2}\cdot s^{-1}$), which is a reasonably accepted factor for sunlight conditions [59].

Photosynthetically Active Radiation (PAR) is one of the most important parameters studied in plant biology. It affects plant growth as plants are highly dependent on that spectrum of light to perform photosynthesis. The amount of PAR absorbed by leaves in different layers within the canopy, strongly influences the amount of carbon fixed per unit ground area, thus making PAR sensors especially useful to assess light conditions in the canopy. Other sensors, such as the Pyranometers, were designed to capture a broader spectrum of radiation, including UV, NIR and thermal, which can also affect plant growth. Together, such sensors can cover the wavelengths important for plant growth, but they can be quite expensive. Therefore, we included a more affordable, simple photo-diode sensor with two spectral responses in this study (see Figure 9). Ultimately, our goal is to measure broad-spectral light exposure and investigate its correlation with growth of different plant genotypes. Here, however, we will limit the discussion to the accuracy of such measurements and present the correlation between data collected from different sensors, in particular the correlations between the inexpensive photo diodes and the more expensive PAR and Pyranometer sensors.

**Figure 10:**
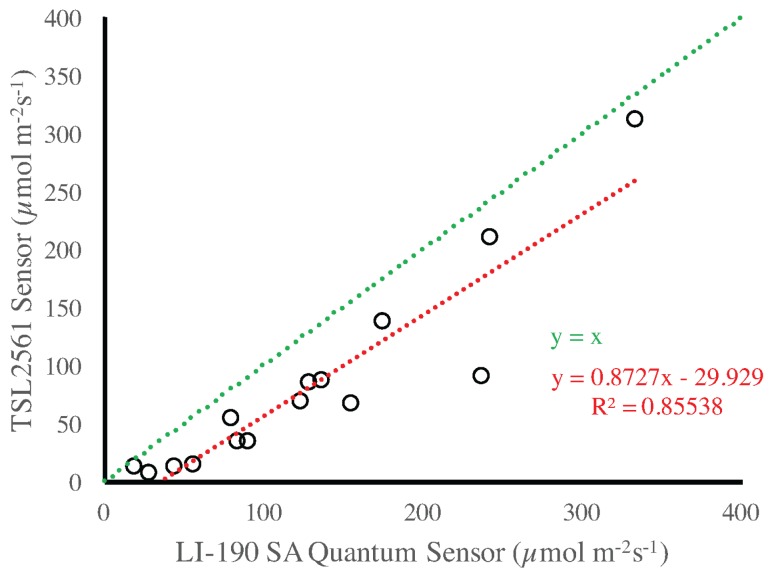
Comparison between data collected with the TSL2561 and the LI-190SA PAR Quantum sensors.

**Figure 11:**
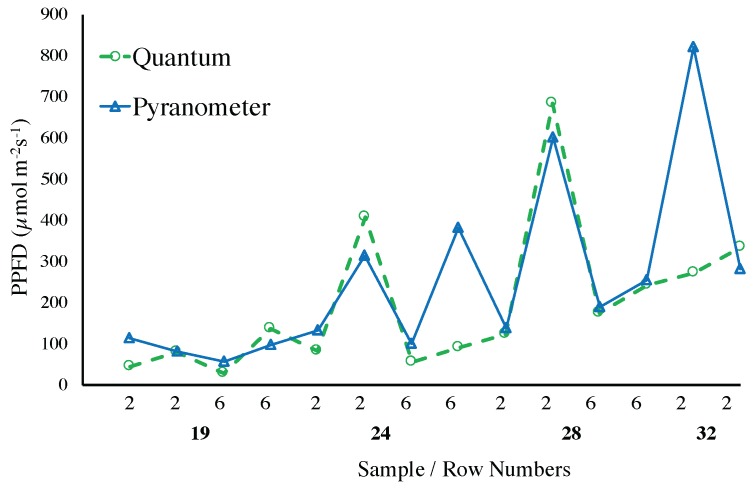
Comparison between the Pyranometer LI-200 SA and the PAR Quantum LI-190 SA. The data is organized over rows of plants, with the horizontal axes showing the number of samples collected per row and the row number from Figure 5.

Figure 10 presents a comparison between the LI-190 SA PAR Quantum sensor and the TSL2561 photo diodes. As expected, for small amounts of light the difference between the two sensors is quite small and the TSL2561 can be a good estimator of PAR. Again, this can be explained by the fact that such measurements are performed mostly in the afternoon, when the IR radiation is greatly reduced. A similar observation can be drawn from Figure 11, where the Pyranometer LI-200 SA and the PAR Quantum LI-190 SA sensors are compared. The reader should be made aware that the data in that Figure is organized over rows of plants just for convenience and in reference to Figure 5.

### 4.2. Vinoculer

As with the Vinobot, the Vinoculer was also used to collect a set of measurements and derived data. These data included a full 3D Models of the plants and determination of LAI—the Vinoculer also gathered air temperature, which will be presented in Section 4.3.

#### 4.2.1. RGB to IR Camera Calibration

As mentioned earlier, the Vinoculer observes the field, collecting both stereo images (for 3D reconstruction) and IR images (for inspection of leaf and soil temperatures). In order to combine the stereo images with the IR image—i.e., to have the correct association between the measurement of the temperature and the reconstructed 3D model of a plant—a calibration procedure is required. Most calibration algorithms developed to date rely on black-and-white chessboards and finding the corners of its squares to derive a calibration matrix relating world and pixel coordinates. However, such corners on regular black and white chessboards cannot be reliably perceived by IR cameras. Thus, we developed a calibration pattern using a multi-material surface: i.e., thin black painted Aluminum squares on top of white paper. This combination of highly heat-absorbent and highly heat-reflective materials created enough contrast for detecting corners and producing an accurate calibration—even on cloudy days.

**Figure 12:**
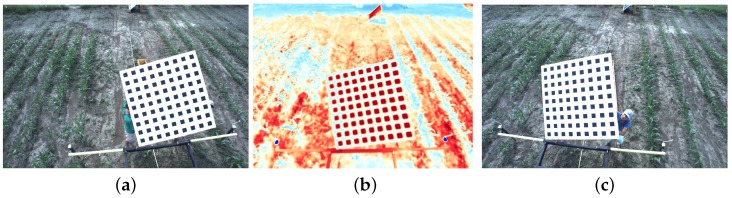
Three typical images of the aluminum + paper pattern used for calibration of RGB and IR cameras. (**a**) Left RGB; (**b**) IR Camera; (**c**) Right RGB.

**Figure 13:**
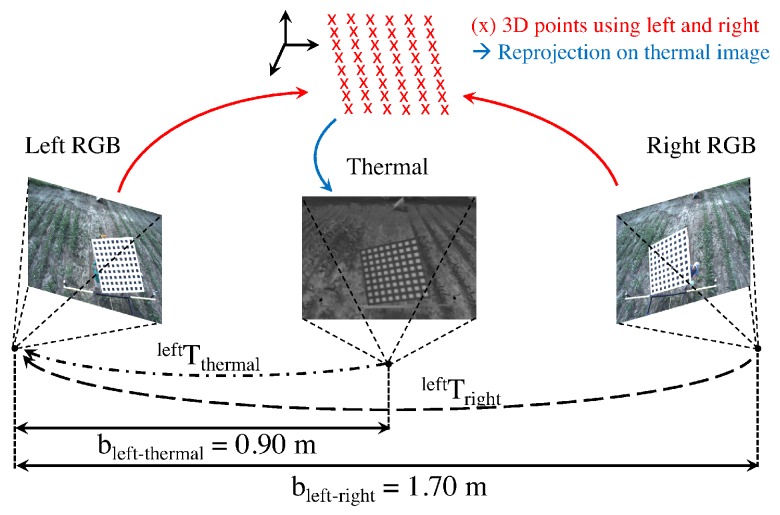
Stereo RGB-Thermal camera configuration.

The developed pattern measured 1.5 m(H)× 1.5 m (W), with 81 black aluminum squares (8×8 ${\mathrm{cm}}^{2}$ each). The black aluminum and white paper squares were applied to a foam board and mounted on a sturdy, but light wooden frame to provide support for the foam-board and two handles on the back—allowing for easy positioning of the pattern during calibration. A popular camera calibration algorithm [60] and its widely available implementation [61] were used to calibrate all three cameras with images captured at 10 different positions and orientations of the calibration pattern. Figure 12 shows three of those images, one for each of the cameras being calibrated. From this calibration process we obtain a set of camera parameters: (1) the intrinsic parameters $K_{left}$, $K_{right}$, $K_{thermal}$; (2) the lens distortion $d_{left}$, $d_{right}$, $d_{thermal}$; and (3) the extrinsic parameters Trightleft, Tthermalleft, which represents the position and orientation of the right and the thermal cameras with respect to the left, or reference camera. Figure 13 illustrates the spatial relationship between the cameras, with the corresponding extrinsic parameters and calibrated baselines indicated.

**Figure 14:**
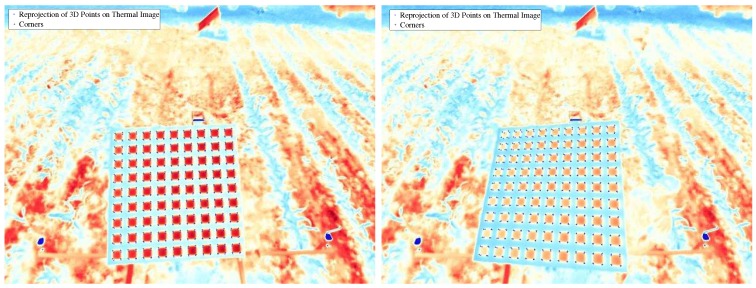
Re-projections of the 3D coordinates of the corners obtained from left-right stereo reconstruction onto the thermal images. The “x” indicate the re-projections, and “+” the extracted corners from the thermal image.

**Figure 15:**
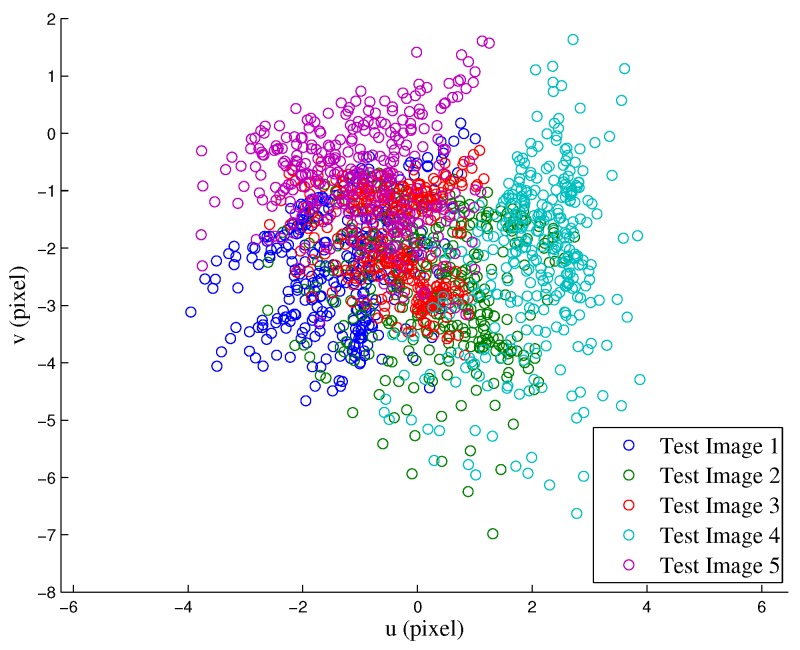
Re-projection error of 3D points found by triangulation of corners on left and right RGB images.

To validate the calibration, the calibration pattern was repositioned and 5 additional sets of images were obtained for testing. The validation was conducted using the following procedure: (1) 3D reconstruction of the corners using left and right RGB images; (2) re-projection of the 3D coordinates of the corners onto the thermal image; and (3) comparison between the re-projected 3D corners and the extracted corners from thermal images. The rationale for this procedure is to encompass all possible sources of error, i.e., from calibration itself, 3D reconstruction, re-projection, and even corner extraction—all of which originated from the combination of right and left cameras, as well as thermal and left (reference) cameras. Both qualitative and quantitative results are reported here in Figure 14 and Figure 15, respectively. From the latter, we calculated an RMS error of 2.57 pixels—with a clear bias towards the vertical direction, which can be removed for more accurate 3D reconstruction.

#### 4.2.2. Traits from 3D Reconstruction

As before, 3D reconstruction was applied to the pair of RGB images, this time from the Vinoculer. The raw images and the 3D models obtained are also publicly available at [53]. Many other traits can be extracted from these high-resolution images, however, for this research we limited the images to 4Mpixels and the extracted features to plant height—measured by the height of the uppermost leaf collar—and Leaf Area Index (LAI). These two measurements were compared with the ground truth from manual phenotyping.

#### 4.2.3. Plant Height

**Figure 16:**
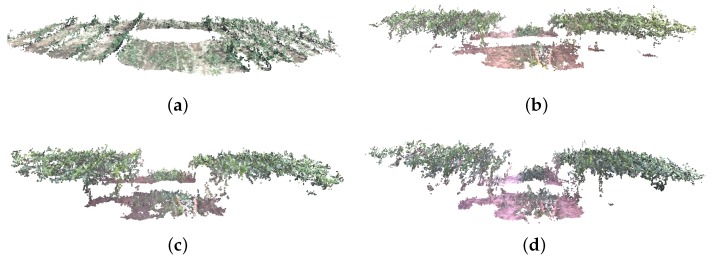
3D Reconstruction of entire field (side view) using stereo RGB images collected by the Vinoculer. (**a**) 23 DAP; (**b**) 36 DAP; (**c**) 39 DAP; (**d**) 45 DAP.

As mentioned earlier, high-throughput phenotyping of groups of plants can be obtained by airborne canopy characterization. In that sense, the Vinoculer is an excellent platform to perform uninterrupted data collection. Based on Vinoculer images, 3D models of the entire field were created to estimate height of the canopy from the average heights of plants within a plot. Figure 16 depicts some of the generated 3D models (side view of the field), for different times during the growing season. From these models, growth rate could also be calculated, and biotic and abiotic stresses could hence be investigated.

**Figure 17:**
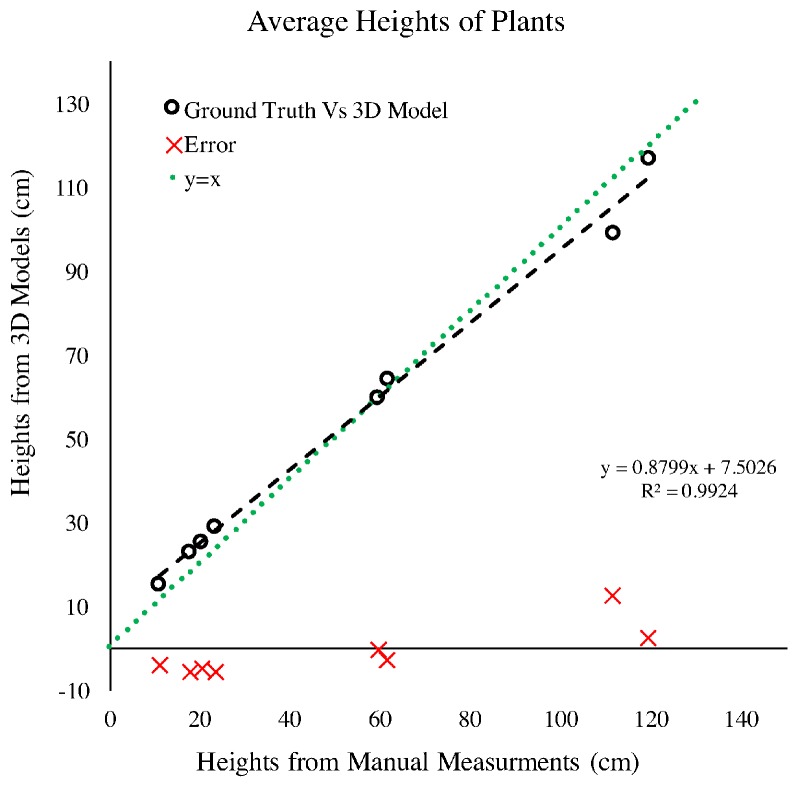
Comparison between manual measurements (Ground Truth) and 3D model based plant height at several time points during the growing season. The 3D model was created using RGB images captured by the Vinoculer.

**Figure 18:**
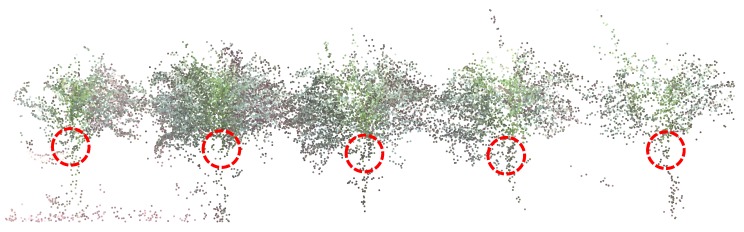
Configuration of selected points as collar of top most leaf on 3D model created by Vinoculer images.

In order to validate our system, the averages of the heights of plants from many plots over multiple days was computed using the 3D models and compared to the ground truth data. Figure 17 shows the relationship between these measurements and their associated errors. The mean square error was calculated to be 2.36 cm. The heights were measured by averaging, among the plants in a same plot, the distances from the collar of the top leaf to the ground—see Figure 18. Since the 3D models from the Vinoculer are not as accurate as those resulting from images obtained by the Vinobot, greater errors are expected in those estimations.

#### 4.2.4. LAI Estimation

Leaf area index was estimated from Vinoculer images using an equivalent to airborne canopy photography by defining a new estimate of gap fraction for 3D images. That is, gap fraction is usually calculated as the ratio between number of background pixels and the total number of pixels within the region of interest (ROI) of the image taken vertically downwards. In order to find background pixels, a segmentation algorithm is employed to distinguish background (soil) and foreground pixels (leaves). LAI is then commonly estimated from vertical gap fraction as [62]:

$$\mathrm{LAI}=-2ln(P_{0})$$

where $P_{0}$ is the vertical gap fraction.

It is important to mention here that this approach to LAI estimation is very error prone, especially when weeds are present. So, in our system, LAI was estimated by first thresholding plants from the 3D model of the field. That is, instead of using a simple foreground and background segmentation based on color, our algorithm also took into account the heights of the point clouds. Since the presence of weeds resulted in a distinct height distribution, our height thresholding approach drastically and more accurately improved the segmentation. Once the foreground (real crop) was segmented from background (soil), a 3D to 2D orthogonal re-projection was applied to the point clouds to create simulated top-down views of the canopy. Finally, from knowledge of the ROIs, vertical gap fractions were calculated using the traditional method discussed above.

**Figure 19:**
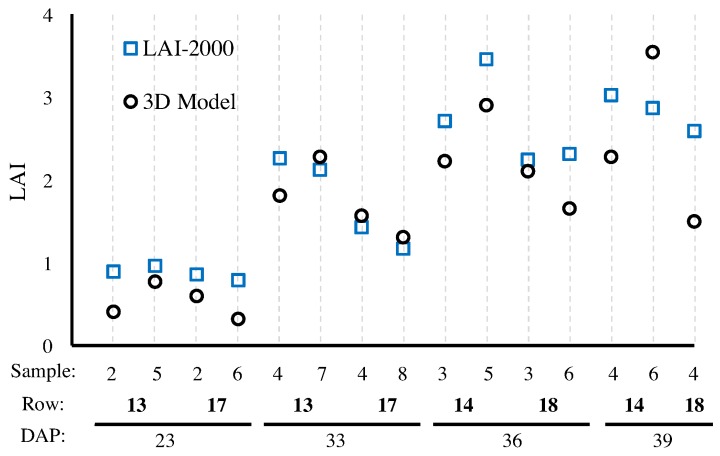
Comparison of LAI estimation between LAI-2000 and 3D Model created by Vinoculer stereo images. The horizontal axes shows the number of samples collected per row; the row number according to Figure 5; and DAP.

Figure 19 presents the results for different plots in the field, obtained during this growing season.

### 4.3. Environmental Data

In this final experiment, environmental data collected by both the Vinobot and the Vinoculer were compared with each other and with the ground truth. As mentioned in Section 3.1.1, three sets of environmental sensors (temperature, humidity and three types of light sensors) were mounted at different heights on a vertical bar and measured near each of the plants phenotyped by the Vinobot. Here, we present the results for the air temperatures near the plants collected by the Vinobot using such vertical bar and the actual plant temperatures registered by the Vinoculer using the IR camera.

#### Temperature

**Figure 20:**
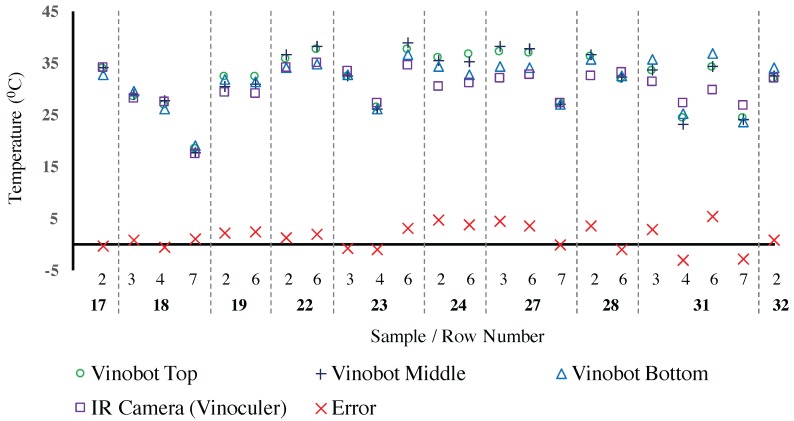
Correlation between the Vinobot temperature sensors and the Vinoculer IR camera.

Even though the two platforms measure different things—i.e., air temperature vs. plant temperature—for the purpose of illustrating their correlation, we compared the measurements from the Vinobot versus the Vinoculer. These measurements are summarized in Figure 20 for different rows of plants, usually obtained on different days. Also, multiple measurements were conducted for each row and for each plant using the different Vinobot sensors at different heights, and the values were averaged for the plot (10 plants). As for the Vinoculer the values were averaged for all IR pixels inside the bounding box representing the sampling plot. Despite the different natures of temperatures, it is interesting to notice that in reference to the field configuration given in Figure 5, the further the row (i.e., the measurement) is from the tower (i.e., from the IR camera), the greater the error. But still, the overall RMS error is less than 1 °C.

**Figure 21:**
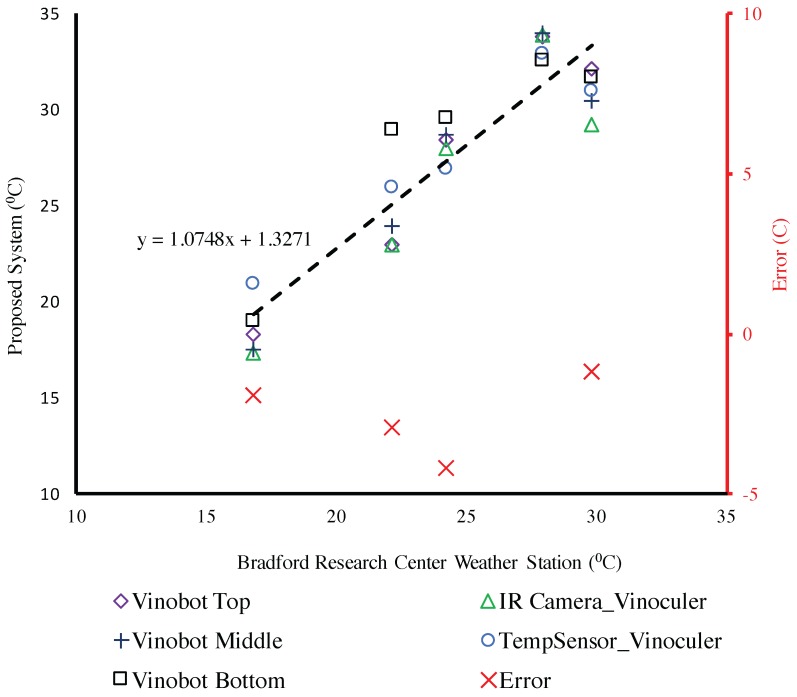
Comparison between temperature measured by proposed system and Ground Truth (Bradford Weather Station [63]).

Finally, we performed a comparison between all temperature sensors available: the three air temperature sensors on Vinobot, the IR camera on Vinoculer, and the air temperature sensor on Vinoculer. Figure 21 presents the correlation between these temperature data and the ground truth for air temperature taken from Bradford Research Center Weather Station [63]. Once again, despite the different nature of those temperatures, it is interesting to notice that a linear regression model drawn by averaging all data for each type of sensor presents a fairly small deviation from the y=x line, with the average RMS error less than 5 °C.

## 5. Conclusions and Future Work

We presented a new approach to high-throughput field phenotyping using two robotic platforms: a ground vehicle and an observation tower. The proposed platforms facilitate phenotyping with spatial and/or temporal resolution that is much greater than what can be achieved with traditional UGV + airborne approaches. But more importantly, the proposed platforms were able to collect data at different scales and by inspecting individual plants as well as the entire field. The high correlation and low errors between these autonomously collected data and the manual measurements demonstrated the accuracy and potential of the proposed platforms.

The 3D models at the plot or individual plant level will allow the development of many other algorithms, and the extraction of additional traits for future comparison with the manually obtained phenotypes. That is, besides the higher volume and higher accuracy when compared to manual measurements, the use of 3D models from both platforms will open the door for in-depth analyses of architectural characteristics, expanding the more traditional measurements of leaf angles, leaf areas, number of leaves, etc into the potential discovery of new traits. Further, the expected fully autonomous deployment of Vinobot for the next season, will allow for the collection of an even greater volume of data while exploiting the use of Vinoculer to direct Vinobot to investigate individual plants in great detail.

## Figures and Tables

**Table 1:** Cost comparison between Vinoculer and 8 UAVs surveyed. The prices do not include any phenotyping sensor.

Platform	Cost (US$)
Vinoculer	5 K
UAV	16–80 K

**Table 2:** Comparison between Vinoculer and the UAV-based phenotyping system presented in [49].

Platform	Payload	Flight Time	Area/Flight	Availability	Total Area Covered	Type of Camera	Max Wind Speed
Phenocopter [49]	1.5 kg	30 min	3 ha	7 flights/day	21 ha/day	RGB, Thermal and NIR	11 m/s
Vinoculer	20 kg	-	-	24/7	2.2 ha/day	Limited by payload	22.3 m/s ^1^

^1^ Recorded wind speed during deployment. Actual maximum wind speed may be greater than that.

**Table 3:** Comparison between field-based systems that perform phenotyping of groups of plants.

Platforms	Type	Plants/h	ha/h	Images/h	Bytes/h	Main Capabilities
Vinobot	Semi-automated	35,430	0.41	324,000	380 G	RGB, temperature, humidity, and light intensity
Vinoculer	Fully-automated	12,648	0.09	2592	5.4 G	Mobile, 24/7, Stereo RGB and IR imaging, Air Temperature
“Phenomobile” [14]	Manually-driven	-	0.84	-	2.094 M	IR, multi-spectral imaging, and sonar sensors
${\mathrm{Scanalyzer}}^{Field}$ [9]	Fully-automated	-	0.002	115	6.7 G	Confined, 24/7, RGB, Multispectral, Fluorescence intensity imaging

**Table 4:** Comparison between field-based systems that perform detailed phenotyping of individual plants.

Platforms	Type	Plants/h	Images/h
Vinobot	Outdoor/Semi-automated	120	1440
${\mathrm{Scanalyzer}}^{3D\;HT}$ [16]	Indoor/Fully-automated	25	375
Phenoscope [13]	Indoor/Fully-automated	185	3750

**Table 5:** Sampling method performed per indicated type of plots.

Plot Type vs. Sampling Method	Manually	Vinoculer	Vinobot
30″ (76 cm) Rows	√	√	
45″ (114 cm) Rows	√	√	√
60″ (152 cm) Rows	√		√
